# BOLD fMRI responses to amplitude-modulated sounds across age in adult listeners

**DOI:** 10.1162/imag_a_00238

**Published:** 2024-07-17

**Authors:** Søren A. Fuglsang, Jonatan Märcher-Rørsted, Kristoffer H. Madsen, Ditte H. Frantzen, Gerard Encina-Llamas, Charlotte Sørensen, Tim B. Dyrby, Torsten Dau, Jens Hjortkjær, Hartwig R. Siebner

**Affiliations:** Danish Research Centre for Magnetic Resonance, Centre for Functional and Diagnostic Imaging and Research, Copenhagen University Hospital—Amager and Hvidovre, Copenhagen, Denmark; Hearing Systems Section, Department of Health Technology, Technical University of Denmark, Kgs. Lyngby, Denmark; Department of Applied Mathematics and Computer Science, Technical University of Denmark, Denmark, Kgs. Lyngby, Denmark; Copenhagen Hearing and Balance Center. Copenhagen University Hospital—Rigshospitalet, Copenhagen, Denmark; Faculty of Medicine, University of Vic—Central University of Catalonia, Vic, Catalonia, Spain; Department of Neurology, Copenhagen University Hospital—Bispebjerg and Frederiksberg, Copenhagen, Denmark; Institute for Clinical Medicine, Faculty of Health and Medical Sciences, University of Copenhagen, Copenhagen, Denmark

**Keywords:** fMRI, age-related effects, auditory, envelope, amplitude modulation

## Abstract

Age-related alterations in the auditory system have been suggested to affect the processing of temporal envelope amplitude modulations (AM) at different levels of the auditory hierarchy, yet few studies have used functional magnetic resonance imaging (fMRI) to study this noninvasively in humans with high spatial resolution. In this study, we utilized sparse-sampling fMRI at 3 Tesla (3T) to investigate regional blood oxygenation level-dependent (BOLD) responses to AM noise stimuli in 65 individuals ranging in age from 19 to 77 years. We contrasted BOLD responses to AM noise stimuli modulated at 4 Hz or 80 Hz with responses to unmodulated stimuli. This allowed us to derive functional measures of regional neural sensitivity to the imposed AM. Compared with unmodulated noise, slowly varying 4 Hz AM noise stimuli elicited significantly greater BOLD responses in the left and right auditory cortex along the Heschl’s gyrus (HG). BOLD responses to the 80 Hz AM stimuli were significantly greater than responses to unmodulated stimuli in putatively primary auditory cortical regions in the lateral HG. BOLD responses to 4 Hz AM stimuli were significantly greater in magnitude than responses to 80 Hz AM stimuli in auditory cortical regions. We find no discernible effects of age on the functional recruitment of the auditory cortex by AM stimuli. While the results affirm the involvement of the auditory cortex in processing temporal envelope rate information, they provide no support for age-related effects on these measures. We discuss potential caveats in assessing age-related changes in responses to AM stimuli in the auditory pathway.

## 1 Introduction

Many older listeners with normal or near-normal audiometric thresholds face challenges in comprehending speech in noisy environments (Gates & Mills, 2005;Humes et al., 2012;Peelle & Wingfield, 2016). It has been proposed that age-related changes in temporal envelope processing at later stages of the auditory system contribute to these difficulties (Erb et al., 2020;Gordon-Salant et al., 2006;Overton & Recanzone, 2016;Pichora-Fuller & Souza, 2003;Pichora-Fuller et al., 2007;Robert Frisina and Frisina, 1997;Snell et al., 2002;Walton et al., 2002). This notion is partly driven by psychoacoustic studies reporting age-related effects on listening tasks where temporal envelope cues play a crucial role in performance (Gordon-Salant et al., 2006;Regev et al., 2023;Snell & Frisina, 2000;Snell et al., 2002;Strouse et al., 2000), although several psychoacoustic studies find no clear support for age-related effects on such measures (Moore et al., 1992;Paraouty & Lorenzi, 2017;Paraouty et al., 2016;Schoof & Rosen, 2014).

Several studies have employed AM sound stimuli to investigate whether neural correlates of temporal envelope processing change with age (Anderson & Karawani, 2020;Parthasarathy et al., 2019;Walton, 2010). Age-related changes in neural synchronization and overall firing rate in response to AM stimuli have been observed at different stages of the auditory system in rodent models (Bureš et al., 2021;Herrmann et al., 2017;Schatteman et al., 2008;Walton et al., 2002) and in auditory cortical regions in rhesus macaque monkeys (Ng & Recanzone, 2018;Overton & Recanzone, 2016).Overton and Recanzone (2016)found that neurons in primary auditory cortex (A1) in aged rhesus macaques exhibited increased spontaneous and evoked firing rates in response to AM noise stimuli compared with younger monkeys. The authors also observed that fewer neurons in the aged monkeys synchronized to AM noise stimuli at modulation rates below 32 Hz, and that young monkeys had more neurons in A1 that responded to AM noise with a change in both the firing rate code and in temporal code (Overton & Recanzone, 2016). These findings were suggested to reflect a perturbed balance of excitation and inhibition with aging in the central auditory system (Overton & Recanzone, 2016;Recanzone, 2018).

Temporal envelope processing of auditory stimuli has been studied with magnetoencephalography (MEG) and electroencephalography (EEG) in humans.Goossens et al. (2016)presented AM noise stimuli to listeners while recording scalp EEG data from each participant. In that study, increases in relative EEG power at the stimulated AM frequency were observed in older adults compared with younger adults for ~4 Hz AM noise stimuli. At a higher AM stimulation rate of ~80 Hz, the authors also noted an age-related decline in relative EEG power at the stimulation frequency, yet this decline was less prominent.Herrmann et al. (2019)also observed enhanced 4 Hz EEG phase locking in older adults compared with younger adults during 4 Hz AM auditory stimulation. Evoked cortical responses to brief sound stimuli (Alain et al., 2022;Henry et al., 2017;Herrmann et al., 2016,^2019^) and M/EEG measures of synchronization to low-frequency speech envelope features (Decruy et al., 2019;Presacco et al., 2016a,2016b,^2019^) have similarly been reported to increase with the age of participants. However, MEG and EEG cannot readily differentiate between AM synchronization at slow AM rates and long-latency auditory-evoked potentials (Edwards & Chang, 2013). Thus, the precise nature of age-related effects on M/EEG-based measures of synchronization to low-rate AM stimuli remains to be clarified.

Complementing EEG and MEG, BOLD fMRI has also been used to explore BOLD signal correlates of sound envelope processing at different stages of the human auditory hierarchy (Barton et al., 2012;Erb et al., 2019;Giraud et al., 2000;Harms & Melcher, 2002;Hart et al., 2003;Herdener et al., 2013;Leaver & Rauschecker, 2016;Overath et al., 2012;Schönwiesner & Zatorre, 2009). However, while BOLD fMRI has been used in several studies to explore age-related effects on sound-evoked responses in various tasks (Cliff et al., 2013;Du et al., 2016;Erb & Obleser, 2013;Grady et al., 2008,^2011^;Lalwani et al., 2019;Peelle et al., 2010;Profant et al., 2015;Rogers et al., 2020), only a few BOLD fMRI studies have specifically explored age-related effects on temporal envelope processing. This was done in a recent study that explored associations between envelope features extracted from speech stimuli and BOLD fMRI responses in older and younger listeners (Erb et al., 2020). Using models for predicting envelope features from BOLD fMRI, the authors suggested that increasing age is associated with a lower cortical selectivity to temporal envelope rate information.

Here, we explored age-related effects on BOLD fMRI responses to AM noise stimuli in a cohort of 65 healthy listeners. Employing a sparse-sampling fMRI paradigm, we presented monaural narrowband noise stimuli during silent time intervals without scanner noise. Stimuli were either unmodulated or fully modulated noise stimuli. Modulated noise stimuli featured a sinusoidal AM at 4 Hz or 80 Hz. Contrasts between regional BOLD responses to AM noise stimuli and responses to unmodulated noise stimuli were used to derive measures of regional sensitivity of cortical and subcortical auditory regions to the imposed temporal features. This allowed us to test whether age was associated with an altered cortical sensitivity to the imposed AM while better accounting for potential age-related changes in responsiveness to sound stimulation.

## 2 Methods and Materials

### 2.1 Participants

Sixty-five individuals, aged between 19 and 77 years (mean 43.2 ± 18.46; 32 male, 33 female), participated in the fMRI experiment. Three additional individuals (all older than 18 years) underwent MRI, but had their scans terminated—one due to an incidental finding and two due to excessive motion during the scans. These participants were excluded from all subsequent analyses. The fMRI experiment was a revised version of the experiment described inFuglsang et al. (2022). Two of the participants from the current study had also taken part in the previous experiment. Handedness was assessed using a Danish version of the Edinburgh Handedness Inventory (Oldfield, 1971). The study received approval from the ethics committee of the Capital Region of Denmark (reference H-19036566), and all participants provided written informed consent before participating in the study.

### 2.2 Pure-tone audiograms

The participants also took part in experiments at the Technical University of Denmark. These experiments were part of a larger study that will be described in future publications. Data acquisition included pure-tone audiogram measurements using ER-3 insert earphones at octave frequencies between 0.25 and 8 kHz. The audiometric experiments were approved by the ethics committee of the Capital Region of Denmark (reference H-16036391). Pure-tone audiograms were available for all participants at audiometric frequencies of 250 Hz, 500 Hz, 1000 Hz, 2000 Hz, 4000 Hz, and 8000 Hz in both ears. Henceforth, PTA denotes in the current study the average of audiometric thresholds across these frequencies. PTA and age were highly correlated (Pearson correlation coefficient of 0.713). When correlating age with audiometric thresholds individually for each ear and audiometric frequency, Pearson correlation coefficients were consistently greater than or equal to 0.408.

### 2.3 MRI data acquisition

Neuroimaging data were collected on a Siemens Magnetom Prisma 3T scanner at the Danish Research Centre for Magnetic Resonance Imaging (DRCMR) using a 64-channel head coil. Data acquisition comprised two scanning sessions. The first session included functional scans and a structural T1-weighted image. The functional scans used an echo-planar T2*-weighted imaging (EPI) sequence (Moeller et al., 2010) with a repetition time (TR) of 7000 ms, acquisition time (TA) of TA = 1541 ms, flip angle = 90^o^, echo time (TE) of 30 ms, slice thickness: 2.6 mm, slice spacing: 20%, multiband acceleration factor: 2, 46 in-plane interleaved slices, acquisition matrix 78 x 78, covering 202 mm by 202 mm field of view. Fat suppression techniques were not applied in functional task-related scans (Fuglsang et al., 2022;Merrett et al., 2021). Functional scans with stimulus presentation were divided into six runs. Each functional run lasted approximately 7.1 minutes. Additional EPI scans with reversed phase-encoding readout were acquired, both with and without fat suppression, in the first scanning session. A T1-weighted magnetization prepared high-resolution gradient echo (MPRAGE) image was acquired (TR = 2700 ms, TE = 3.7 ms, flip angle = 9^o^, inversion time TI = 1090 ms, voxel size: 0.9 mm x 0.9 mm x 0.9 mm, in-plane acceleration via GRAPPA (Griswold et al., 2002) with a factor of 2 and 24 reference lines). The second scanning session included diffusion-weighted scans, a quantitative mapping protocol, and a high-resolution T2-weighted scan (TR = 3200 ms, TE = 408 ms, flip angle = 120^o^voxel size: 0.9 mm x 0.9 mm x 0.9 mm, in-plane acceleration via GRAPPA with a factor of 2 and 24 reference lines). The sequences and data from the diffusion-weighted and the quantitative mapping protocols will be considered in future publications, and are not described in further detail here. Pulse and respiration data were acquired from participants during functional scans with a respiration belt and a pulse oximeter attached to the left ring finger.

### 2.4 fMRI experiment

Sound stimuli were presented during the silent intervals between acquisitions in the functional runs. Each functional run consisted of 59 scans and 3 initial dummy scans that were excluded from all subsequent analyses. A sparse “blocked” design was considered, where sound stimuli were presented in “blocks” containing two consecutive trials with identical stimulus types (i.e., noises with the same amplitude modulator, as described in the next section), followed by two “silent trials” with no stimulus presentation. Silent trials are illustrated schematically inFigure 1a. Each functional run contained one or two catch trials where the noise stimuli were presented at a lower sound pressure level (SPL). Catch trials were always preceded by a stimulus trial. The order of stimulus blocks was pseudorandomized, subject to the constraint that the first and last stimulus block could not contain catch trials, and that two catch trials could not occur in consecutive blocks. Each functional run comprised at least four blocks for each of the three stimulus types. Two silent trials were included in the beginning and end of each functional scan. Participants were instructed to press a button with their right index finger when they heard sound stimuli presented at a lower sound pressure level (i.e., a catch trial). They were instructed to minimize motion during each scan and to gaze on a crosshair shown visually on a projector screen during functional scans. Due to a technical issue, button presses were not logged for one younger participant who otherwise reported responding to catch trials. However, this participant was not excluded from the subsequent fMRI analyses.

**Fig. 1.**
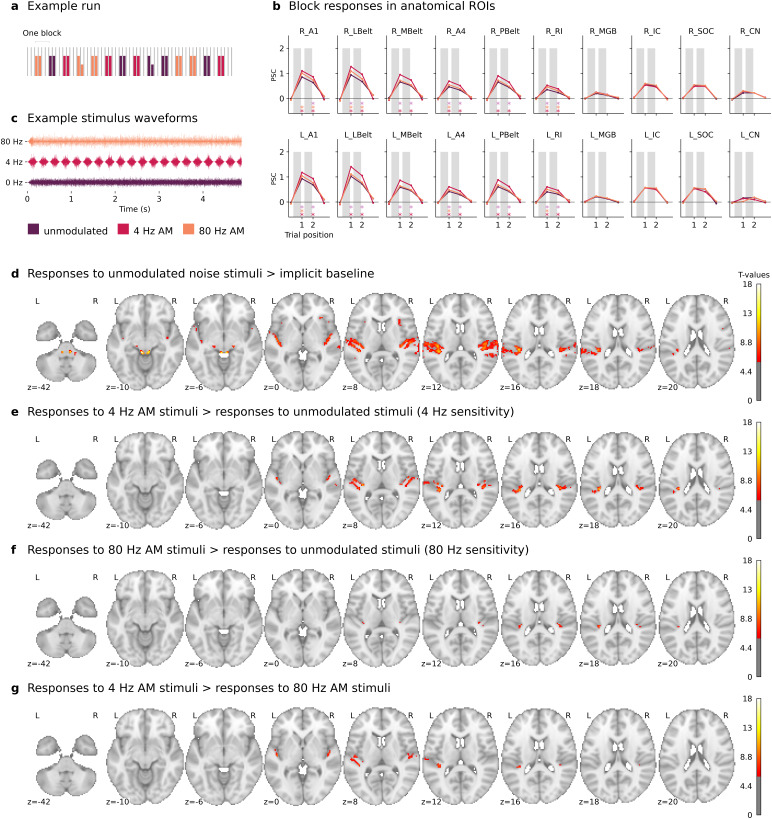
(a) Schematic representation of an experimental run for a given participant. Each functional run consisted of stimulus trials, silent trials, and catch trials. The timing of acquisitions is visualized with light gray vertical bars. The colors are used to indicate different stimulus types. Bars of lower height indicate catch trials. (b) Group-mean responses in auditory regions of interests (ROIs) for the three stimulus types. Each panel reflects average percent signal change (PSC) in a given ROI. The top row shows data from ROIs in the right hemisphere and the bottom row shows data from ROIs in the left hemisphere. Asterisks below each panel are used to indicate significant differences between responses to different stimulus types (purple: 4 Hz vs. 80 Hz, orange: 80 Hz vs. unmodulated, red: 4 Hz vs. unmodulated). Regions of interest include the primary auditory cortex (A1), lateral belt complex (LBelt), medial belt complex (MBelt), retroinsular area (RI), auditory 4 complex (A4), parabelt complex (PBelt), medial geniculate body (MGB), inferior colliculus (IC), superior olivary complex (SOC), and cochlear nucleus (CN). (c) Schematic representation of stimulus waveforms for three realizations of different noise types. Stimuli in stimulus trials were either unmodulated or modulated at 4 Hz or 80 Hz. The different stimulus types are depicted in different colors. (d–g) Whole-brain second-level results. Each panel shows thresholded maps of*t*-statistics (based on maps of family-wise error rate (FWER) corrected*p*-values).

### 2.5 fMRI sound stimuli

Noise stimuli were presented to participants in the 3T Siemens Prisma scanner via Sensimetrics S14 earphones. Unique realizations of Gaussian white noise were used as noise carriers. The stimuli each had a duration of 4.8813 seconds. Unmodulated and amplitude-modulated stimuli were all ramped with 1/8 seconds long (half a 4 Hz cycle) onset ramps and 1/160 seconds long (half an 80 Hz cycle) offset ramps to make the stimulus onset and offset comparable across the different imposed modulations and the unmodulated stimuli. Modulated stimuli had imposed sinusoidal AM with modulation rates of either 4 Hz or 80 Hz. The sinusoidal amplitude modulations were imposed on stimuli in the 4.75 seconds long time periods outside the onset/offset ramps (corresponding to 19 cycles of a 4 Hz modulator and 380 cycles of an 80 Hz modulator). The modulation depth was 100%. Stimuli were high-pass filtered (second order Butterworth filter with a cutoff at 100 Hz), low-pass filtered (ninth order Butterworth filter with a cutoff at 2000 Hz), and linearly equalized to account for the frequency response of the earphones. Stimuli were presented in stimulus trials in the silent interval between two acquisitions. Stimuli in all stimulus blocks had a root mean square SPL of 80 dB SPL. Stimuli in catch trials had a root mean square SPL of 60 dB SPL. The SPL was calibrated using a Norsonic Nor139 sound level meter, a G.R.A.S. 42AP pistonphone and a G.R.A.S. IEC 60318-4 Ear Simulator Kit. Audio stimuli were generated in MATLAB (MathWorks, Natick, MA) version R2018b. Waveforms of example sound stimuli are shown inFigure 1c.

### 2.6 Anatomical data processing

T1w and T2w images were processed with FreeSurfer 7.1.0, using the recon-all processing pipeline (Dale et al., 1999;Fischl et al., 1999,^2002^,^2004^). A study-specific template was created from processed T1w and T2w images using*antsMultivariateTemplateConstruction2*available with Advanced Normalization Tools (ANTs Version 2.3.4) (Avants et al., 2009,^2011^). Spatial normalization to MNI152 was achieved with*antsRegistrationSyN*available with ANTs (Avants et al., 2008,^2011^;Tustison & Avants, 2013).

### 2.7 fMRI data processing

The first two volumes in each functional scan were excluded from subsequent analyses (no stimulus trials were discarded). Functional scans underwent motion correction using SPM12 (Friston et al., 1995). No slice-timing correction was applied due to the sparse-sampling sequence. The tools*TOPUP*and*applyTOPUP*, available with FSL 6.0.1 (Jenkinson et al., 2012;Smith et al., 2004), were used to address geometric distortions in the functional data based on EPI with reversed phase-encoding directions (Andersson et al., 2003). Coregistration between anatomical and functional images was achieved with FreeSurfer tool*bbregister*(Greve & Fischl, 2009). Functional data from each scan were resampled into MNI space using the ANTs tool*antsApplyTransforms*(with Lanczos interpolation). This step also involved Convert 3D (c3d) available with ITK-SNAP. Functional data processing workflows were configured in Python and utilized Nipype (Gorgolewski et al., 2011), along with libraries such as Numpy (Harris et al., 2020), Scipy (Virtanen et al., 2020), and Nibabel (Brett et al., 2023). Preparation of visualizations made use of Seaborn (Waskom, 2021), Nilearn (Abraham et al., 2014), and Matplotlib (Hunter, 2007).

### 2.8 Regions of interest

We considered coarse-grained anatomical regions of interest (ROIs) that had also been considered inFuglsang et al. (2022). Cortical ROIs for A1, lateral belt complex (LBelt), medial belt complex (MBelt), retroinsular area (RI), auditory 4 complex (A4), and parabelt complex (PBelt) from the HCP-MMP 1.0 atlas (Glasser et al., 2016) were considered. We focused on volumetric cortical ROIs in native participant space created using the FreeSurfer tools*mri_surf2surf*and*mri_aparc2aseg.*Subcortical auditory ROIs for the cochlear nucleus (CN), inferior colliculus (IC), superior olivary complex (SOC), and medial geniculate body (MGB), based on ROIs defined bySitek et al. (2019), were further considered. All volumetric ROIs were resampled to the target MNI template using ANTs tools.

### 2.9 First-level analyses

General linear models (GLMs) were employed for participant-specific data analysis using SPM12. The models included sets of regressors for each run, including intercept terms, motion parameters (six regressors for translations in x, y, and z directions and rotations about x, y, and z; one framewise displacement coefficient), and six RETROICOR (Glover et al., 2000) parameters. The analyses incorporated high-pass filters with filter cutoffs of 100 seconds. The models included boxcar regressors flagging catch trials and trials, in which participants had pressed the response button. Boxcar regressors for each stimulus type (unmodulated, modulated at 4 Hz, and modulated at 80 Hz) as well as parametrically modulated stimulus regressors were included in the models. Parametrically modulated stimulus regressors were included to account for potential offsets in response magnitude between the first and second trials within stimulus blocks. Each parametric regressor was orthogonalized with respect to its corresponding stimulus regressor. For simplicity, we chose to model the stimuli as boxcar regressors with additional regressors accounting for difference between the two time points, which is equivalent to modeling the response as a finite impulse response with two bins. We remark that highly similar results were obtained using a model that involved convolution with a standard canonical hemodynamic response function (Perrachione & Ghosh, 2013) as implemented in SPM12. We focused on the following four contrasts of interest: (1) responses to unmodulated noise stimuli against implicit baseline, (2) responses to 4 Hz AM noise stimuli against responses to unmodulated noise stimuli, (3) responses to 80 Hz AM noise stimuli against responses to unmodulated noise stimuli, and (4) responses to 4 Hz AM noise stimuli against responses to 80 Hz AM noise stimuli. We will refer to these as “*responses to unmodulated stimuli,”*“*sensitivity to 4 Hz AM,*” “*sensitivity to 80 Hz AM,*” and “*AM feature preference*,” respectively.

### 2.10 Voxel-level second-level analyses

We conducted whole-brain second-level analyses to explore potential associations between participants’ age and BOLD fMRI measures; specifically, responses to unmodulated stimuli, sensitivity to 4 Hz AM, sensitivity to 80 Hz AM, and AM feature preference. We employed Permutation Analysis of Linear Models (PALM;Winkler et al., 2014) in MATLAB 2018a (MathWorks, Natick, MA). Separate linear models were formulated for each measure, with group-level designs that included age as the regressor of interest and sex and PTA as nuisance variables. The group-level designs included intercept terms. Regressors for age, sex, and PTA were demeaned. Our focus was on voxel-level inference and on the null hypotheses that slopes associated with the age term were equal to 0. Two-sided permutation tests (Holmes et al., 1996;Winkler et al., 2014) with a Freedman–Lane permutation method (Freedman & Lane, 1983) and 10000 permutations were considered. A max-type procedure was used to control family-wise error rate (FWER) and derive adjusted*p*-values corrected across voxels and contrasts (Alberton et al., 2020;Winkler et al., 2016). The MNI152 2 mm brain mask distributed with FSL was supplied as mask for the analyses. The above procedure was repeated with models that excluded PTA from the group-level designs to better understand whether conclusions would change without this variable (see also section “Challenges in BOLD fMRI with different age groups”).

We were further interested in exploring group-level activations related to responses to unmodulated stimuli, sensitivity to 4 Hz AM, sensitivity to 80 Hz AM, and AM feature preference. This was addressed using whole-brain second-level analyses. Here, we repeated the above procedure, focusing on intercept terms, and using sign-flipping procedures in place of permutation procedures.

### 2.11 Associations between age and BOLD fMRI measures in ROIs

As a complementary analysis, we further explored associations between participant age and mean BOLD fMRI contrast estimates in the previously described anatomical ROIs. We focused on the same group-level designs as described previously, and we once again explored group-level designs that either included or excluded PTA as a regressor of no interest. A permutation inference framework like that described in the previous section was considered, and a max-type procedure was used to control FWER across ROIs and contrasts. Note that it is difficult to spatially isolate subcortical ROIs (in particular the CN) due to the low spatial resolution of the EPI sequence and due to partial volume effects.

### 2.12 Block responses to different stimulus types in ROIs

Effects of stimulus type (i.e., unmodulated, 4 Hz AM, or 80 Hz AM) on BOLD fMRI responses in each of the two subsequent stimulus trials in stimulus blocks were examined for each ROI. To this end, we computed the spatial average of voxel time courses across voxels within each ROI for each participant and each functional run. Motion parameters, RETROICOR parameters, and slow trends were projected out from the spatially averaged time courses using linear regression models, followed by adding the temporal mean values to the residualized time courses. These time courses were converted to percent signal change (PSC) by subtracting and dividing time courses by the average across trials with no sound stimuli. For each participant, PSC was averaged across responses to stimulus blocks with the same AM type, excluding blocks with catch trials or blocks where the participant had pressed the response button. Subsequently, we contrasted responses to the different stimulus types, specifically, responses to 4 Hz AM against responses to unmodulated noise, responses to 80 Hz AM against responses to unmodulated noise, and responses to 4 Hz AM against responses to 80 Hz AM. This was done separately for both subsequent stimulus trials in stimulus blocks using two-sided paired*t*-tests. A Bonferroni correction was applied to address multiple testing across ROIs (20 ROIs), stimulus trials (2 consecutive trials), and contrasts (the 3 previously described contrasts), resulting in 120 tests. For the visualization inFigure 2, we computed the average block responses across stimulus repeats and stimulus trials for each participant and each stimulus type.

**Fig. 2.**
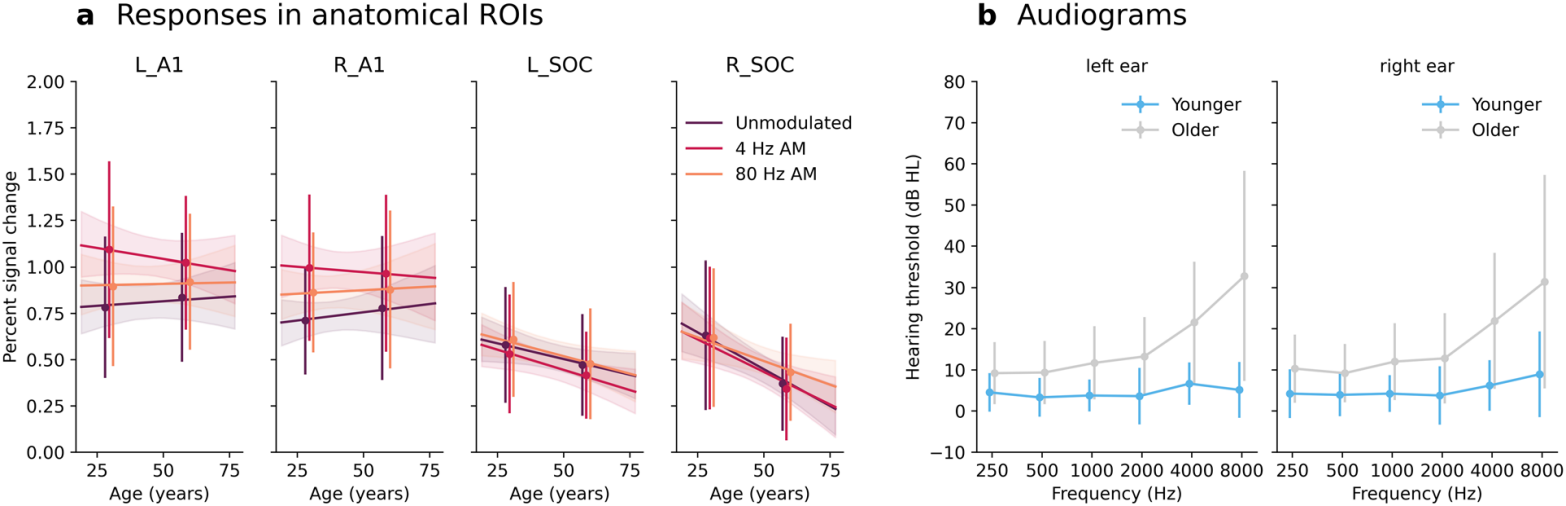
(a) Visualization of responses to the different noise stimuli in the left and right A1, as well as in the left and right superior olivary complex (SOC), plotted against age of the participants. Each panel depicts regression lines derived from linear regression models that each includes an intercept term and age of the participants. Shaded areas represent 95% confidence intervals of the regression lines obtained via bootstrapping (10000 iterations). Vertical lines indicate standard deviations after dividing participants into two age groups. The “younger” group comprises data from 33 participants (16 females) aged 40 years or younger, while the “older” group comprises data from 32 participants aged over 40 years (17 females). Notice that this figure divides listeners into two groups for visualization purposes, but that the second-level models included age as a continuous variable. (b) Audiograms for each ear. Lines represent data from the “younger” (light blue) and the “older” (light gray) groups, with error bars representing standard deviation. Error bars have been adjusted around the center frequency for improved visualization clarity.

## 3 Results

### 3.1 Whole-brain analyses of response differences across amplitude modulators

Group analyses were used to examine sensitivity to 4 Hz and 80 Hz amplitude modulations, AM feature preference, as well as responses to unmodulated noise stimuli. Relative to the implicit baseline, unmodulated noise generated significant group-level activations across the auditory pathway, encompassing auditory cortices and subcortical structures corresponding to SOC, IC, and MGB (Fig. 1d). When compared with unmodulated noise stimuli, slowly varying 4 Hz AM noise stimuli exhibited significantly greater BOLD responses in auditory cortices along HG (Fig. 1e; FWER-correction,α=0.05, two-sided tests), without a similar effect in any subcortical region. This aligns with our previous findings (Fuglsang et al., 2022). BOLD fMRI responses to the faster 80 Hz AM stimuli were significantly greater than responses to unmodulated stimuli in putatively primary auditory cortical regions in the lateral HG (Fig. 1f; FWER correction,α=0.05, two-sided tests). When compared with 80 Hz AM noise stimuli, slowly varying 4 Hz AM noise stimuli exhibited significantly greater BOLD fMRI responses in auditory cortices along HG (Fig. 1e; FWER-correction,α=0.05, two-sided tests).

### 3.2 Block responses to different AM stimuli

Figure 1billustrates group-averaged BOLD fMRI responses to the three different stimulus types in each ROI. Responses to 4 Hz AM noise stimuli were significantly greater than responses to unmodulated noise in both stimulus trials in all the considered cortical ROIs (two-sided paired*t*-tests,p<0.05​/​120). Responses to 80 Hz AM noise stimuli were greater than responses to unmodulated noise in the first stimulus trial in the left and right A1, A4, and RI as well as in the right LBelt (two-sided paired*t*-tests,p<0.05​/​120). Furthermore, responses to 80 Hz AM were significantly greater than responses to unmodulated noise in the second stimulus trial in the right RI, in the right A1, and in the right LBelt (two-sided paired*t*-tests,p<0.05​/​120). When comparing responses to the different AM noise stimuli, we found that 4 Hz AM noise elicited greater responses than 80 Hz AM noise in both stimulus trials in all of the considered cortical ROIs, except in the right A1 where 4 Hz AM noise was found to elicit significantly stronger responses than 80 Hz AM noise in only the second stimulus trial (two-sided paired*t*-tests,p<0.05​/​120).

### 3.3 Associations between age and BOLD fMRI measures

Next, we investigated associations between age of the participants and BOLD fMRI contrast estimates for each of the four contrasts of interest (responses to unmodulated noise, sensitivity to 4 Hz AM, sensitivity to 80 Hz AM, and AM feature preference). Whole-brain second-level analyses did not reveal any significant voxel-level age associations for either of these contrasts (FWER-correction,α=0.05, two-sided tests). This analysis was repeated for mean contrast estimates in the anatomical ROIs. Once again, no significant associations were observed between the age of the participants and mean contrast estimates in anatomical ROIs for either of the four contrasts (FWER-correction,α=0.05, two-sided tests).

These analyses were repeated using second-level models that did not include PTA as a regressor in the group-level designs. Whole-brain second-level models that did not include PTA as a regressor did not reveal any significant age associations for either of the contrasts (FWER-correction,α=0.05, two-sided tests). Excluding PTA as a regressor in second-level models of data in ROIs did not change any of the main conclusions, with one exception: when excluding PTA, there was a significant negative age slope for responses to unmodulated noise in the right SOC (p<0.05, FWER corrected, two-sided test). As a visual reference point,Figure 2provides an illustration of responses to the different noise stimuli in the left and right A1, and in the left and right SOC. Notice that this figure divides listeners into two groups for visualization purposes.

## 4 Discussion

Our sparse-sampling fMRI approach suggested a functional involvement of the auditory cortex in processing of temporal envelope rate information. No sensitivity to either 4 Hz or 80 Hz AM could be observed in subcortical auditory regions. Despite these observed associations between regional BOLD responses in auditory cortex and the AM stimuli, we found no significant associations between the age of the participants and regional sensitivity to AM in auditory cortex within the studied age range.

### 4.1 Comparison with previous M/EEG studies

We found that slowly varying 4 Hz AM noise stimuli elicited significantly greater BOLD responses in the left and right auditory cortices along Heschl’s gyrus (HG) compared with unmodulated noise. The response sensitivity to 4 Hz AM in auditory cortical regions is consistent with findings from previous fMRI studies (Fuglsang et al., 2022;Overath et al., 2012). Similarly, BOLD responses to the faster 80 Hz AM stimuli were notably greater than responses to unmodulated stimuli in putatively primary auditory cortical regions in the lateral HG. Responses to slowly fluctuating 4 Hz AM stimuli were found to be significantly greater than responses to 80 Hz AM stimuli in the left and right auditory cortices along HG, a finding that aligns well with previous BOLD fMRI studies that also suggest heightened preference for slow AM features in auditory cortical regions (Erb et al., 2019;Overath et al., 2012;Santoro et al., 2014).

Several recent human M/EEG studies have reported increased cortical synchronization to slow AM with pronounced 4 Hz AM in older listeners compared with younger listeners (Decruy et al., 2019;Goossens et al., 2016,^2019^;Herrmann et al., 2019;Presacco et al., 2016a,2016b). Using fully modulated sinusoidal AM stimuli,Goossens et al. (2016)found that auditory steady-state responses (ASSRs) to ~4 Hz AM noise stimuli were increased in older adults compared with younger adults, all with audiometric thresholds ≤25 dB hearing level (HL) at octave frequencies from 125 Hz up to 4 kHz. These age-related enhancements align with results fromHerrmann and Johnsrude (2018), who similarly found an enhanced EEG synchronization to sound stimuli with pronounced 4 Hz AM.

The results of the present study remain inconclusive regarding the age-related effects of AM sensitivity to 4 Hz AM features which may appear surprising, considering the previous M/EEG studies that suggest age-related enhancements in M/EEG synchronization to 4 Hz AM stimuli. This discrepancy may relate to differences between BOLD fMRI readouts and M/EEG readouts. In our study, we contrasted BOLD fMRI responses to 4 Hz AM stimuli against responses to unmodulated noise as a measure of sensitivity to 4 Hz AM. The extent to which this measure aligns with M/EEG measures of 4 Hz AM synchronization is less clear. For example, in M/EEG responses to repetitive stimuli with slow repetition rates, long-latency auditory-evoked potentials from individual stimulus cycles may overlap and interact, affecting EEG measures of synchronization to the envelope fluctuations (Edwards & Chang, 2013). Potential age effects on evoked responses could also affect M/EEG 4 Hz synchronization measures, complicating their interpretation. Studies such asIrsik et al. (2021), which explore stimuli with different envelope shapes, are intriguing for further illuminating the nature of age-related effects on low-rate EEG synchronization measures. Additional studies are needed to explore the relationships between M/EEG measures of synchronization to AM stimuli and BOLD fMRI measures of AM sensitivity.

Reports on age-related effects of M/EEG response properties at rates around 80 Hz have been somewhat mixed.McClaskey et al. (2019)investigated EEG responses to AM stimuli with pronounced 80 Hz AM at different modulation depths but found no significant effects of age groups on EEG responses.Goossens et al. (2016)observed a small but significant age-related decline in ASSRs to ~80 Hz AM noise stimuli.Gaskins et al. (2019)measured EEG responses to band-limited pulse trains in 15 younger listeners and 15 older listeners (all with normal hearing) and found no significant age effects on ASSR responses to pulse trains with 80 Hz repetition rates. It is plausible that ASSR measures of synchronization to AM with rates around 80 Hz are highly dependent on stimulus details (e.g., spectral sidebands, sound power, or modulation spectra), making it challenging to directly compare results from these different studies. Further studies are needed to clarify effects of age on auditory responses to 80 Hz AM stimuli.

### 4.2 BOLD fMRI measures of sensitivity to AM

Several BOLD fMRI studies in humans have explored BOLD fMRI correlates of AM processing and whether auditory cortex shows preference for distinct AM features (Barton et al., 2012;Giraud et al., 2000;Harms & Melcher, 2002;Hart et al., 2003;Herdener et al., 2013;Leaver & Rauschecker, 2016;Overath et al., 2012;Schönwiesner & Zatorre, 2009). Our results suggested heightened sensitivity to 4 Hz AM features in auditory cortical regions as well as sensitivity to 80 Hz AM stimuli in primary auditory cortices in both hemispheres. Our results further suggest a heightened preference for 4 Hz AM stimuli compared with 80 Hz AM stimuli in auditory cortical regions. However, we found no significant effects of AM on BOLD fMRI responses in any subcortical regions. It is plausible that the coarse spatial and temporal resolution of our BOLD fMRI measurements makes it difficult to derive measures of subcortical envelope processing. We cannot rule out that this explains why we find no subcortical voxels that exhibit significant sensitivity to slow AM, given that earlier fMRI studies (Giraud et al., 2000;Harms & Melcher, 2002,^2003^) indeed suggest AM-related changes in BOLD response waveshape at different stages of the auditory hierarchy.

Other studies (Erb et al., 2019,^2020^;Santoro et al., 2014) use speech stimuli and computational models to derive BOLD fMRI measures of AM processing. These approaches are appealing, but one challenge here is that one must rely on computational models of envelope processing that inherently are oversimplified. Moreover, accounting for potentially confounding auditory features can be challenging in such analyses. For example, auditory cortical regions may exhibit selectivity to semantic features (Huth et al., 2016), phonetic features (Di Liberto et al., 2015;Khalighinejad et al., 2017;Mesgarani et al., 2014), and articulatory features (de Heer et al., 2017), and such feature sets may be correlated with AM features of interest which can complicate inferences (Daube et al., 2019;Fuglsang et al., 2024). Relationships between measures of AM processing derived with synthetic AM stimuli and results from such model-based analyses with speech stimuli remain to be further clarified.

### 4.3 Challenges in BOLD fMRI with different age groups

We explored associations between our BOLD fMRI contrast measures and the age of the participants. This was done using regression analyses that included sex and PTA as regressors of no interest. These analyses revealed no significant associations between BOLD fMRI measures of sensitivity to 4 Hz AM, sensitivity to 80 Hz AM, AM feature preference, or responses to unmodulated noise and the age of the participants. However, a common challenge in BOLD fMRI studies involving individuals of varying age is the potential sensitivity of BOLD readouts to age-related changes in vascular function or brain anatomy (D’Esposito et al., 2003;Tsvetanov et al., 2021). This complexity also applies to the present study, making it challenging to disentangle such effects from those associated with changes in neural processing.

The present study utilized a sparse-sampling fMRI scanning protocol, allowing for the presentation of AM stimuli during periods without scanner acquisition noise. However, sparse sampling may result in physiological noise signals not being critically sampled, complicating the adequate removal of physiological noise from the data. Additionally, our sparse-sampled fMRI data do not permit the direct inference of BOLD response waveforms to the AM stimuli or the assessment of potential age-related changes in the temporal pattern of the BOLD response waveforms. Future studies are needed to explore whether the temporal dynamics of hemodynamic response waveforms to AM sounds exhibit age-related changes.

### 4.4 Hearing sensitivity in individuals with different age spans

A complicating factor in most studies of auditory temporal envelope processing involving listeners of different age spans is the tendency for hearing thresholds to be elevated in older listener groups. It can be challenging, if not impossible, to recruit older participants with audiograms matching those of young normal-hearing participants, in particular with extended high-frequency audiograms. This opens for the possibility that threshold losses in older listeners affect correlates of neural responses to the presented suprathreshold sounds. For the same reason, many studies (including ours) focus on narrowband sound stimuli confined to lower frequencies and hope that typical age-related audibility losses at higher frequencies outside the stimulus frequency range do not impact responses. However, threshold elevations at extended high frequencies (>8 kHz) can affect neural responses even when the threshold elevations are beyond stimulation frequencies of the noise stimulus (<2 kHz) due to the level-dependent upward spread of excitation in the cochlea. Threshold elevations can also impact perception of suprathreshold sound stimuli, such as perceived loudness. Age and hearing sensitivity are typically highly correlated (also at lower audiometric frequencies), even after excluding listeners based on a cutoff threshold for what is considered a clinically “normal” audiogram. There is currently no consensus on whether measures of hearing sensitivity should be included in statistical analyses in age studies that aim to characterize neural responses to sounds presented at fixed SPLs. For example,Carcagno and Plack (2022),Leung et al. (2013), andMcClaskey et al. (2019)included hearing threshold measures as variables in their statistical models, whereasErb et al. (2020),Henry et al. (2017), andHerrmann et al. (2016)did not. This discrepancy potentially reflects that there is no clear agreement on assumed causal relationships between age, hearing sensitivity, and neural responses to sounds in these scenarios. Another complicating factor is that PTA and other measures derived from audiometric thresholds may be poor descriptors of stimulus audibility reductions. For transparency, we explored whether conclusions regarding the age associations would change if we excluded PTA as a variable in our regression analyses. This was not the case, with only one exception: when excluding PTA, there was a significant negative association between age and BOLD fMRI responses to unmodulated noise in the right SOC. Future studies should clarify the influences of hearing threshold elevations on age-related measures of temporal processing.

## 5 Concluding Remarks

We examined relationships between age and BOLD fMRI responses to unmodulated noise or amplitude-modulated noise stimuli. Our results indicated that BOLD fMRI responses to 4 Hz AM stimuli were generally higher than responses to unmodulated noise stimuli in auditory cortical regions. Additionally, responses to 4 Hz AM stimuli were significantly greater than responses to the 80 Hz AM stimuli in auditory cortical regions, while responses to 80 Hz AM stimuli surpassed those to unmodulated noise in the bilateral auditory cortex. The heightened auditory cortical sensitivity to slow 4 Hz AM—a temporal AM feature that tends to be prominent in speech (Poeppel and Assaneo, 2020)—aligned with previous findings (Fuglsang et al., 2022;Overath et al., 2012). However, we observed no significant associations between age and our BOLD fMRI contrast measures, neither in whole-brain analyses nor in analyses in anatomical ROIs. We cannot rule out that this may be attributed to the sensitivity of BOLD fMRI readouts to other factors affecting the signal-to-noise ratio, or to the constraints imposed by our limited sample size.

## Data Availability

The data used for this study are part of an ongoing investigation and are not publicly available. Code is available upon reasonable request.
